# COVID-19 Misinformation and Social Network Crowdfunding: Cross-sectional Study of Alternative Treatments and Antivaccine Mandates

**DOI:** 10.2196/38395

**Published:** 2022-07-27

**Authors:** Nathan M Shaw, Nizar Hakam, Jason Lui, Behzad Abbasi, Architha Sudhakar, Michael S Leapman, Benjamin N Breyer

**Affiliations:** 1 Department of Urology University of California, San Francisco San Francisco, CA United States; 2 Department of Urology Yale University School of Medicine New Haven, CT United States; 3 Department of Epidemiology and Biostatistics University of California, San Francisco San Francisco, CA United States

**Keywords:** COVID-19, misinformation, infodemic, social media, GoFundMe, vaccine hesitancy, vaccination, infodemiology, campaign, treatment, vaccine mandate, health care, online health information

## Abstract

**Background:**

Crowdfunding is increasingly used to offset the financial burdens of illness and health care. In the era of the COVID-19 pandemic and associated infodemic, the role of crowdfunding to support controversial COVID-19 stances is unknown.

**Objective:**

We sought to examine COVID-19–related crowdfunding focusing on the funding of alternative treatments not endorsed by major medical entities, including campaigns with an explicit antivaccine, antimask, or antihealth care stances.

**Methods:**

We performed a cross-sectional analysis of GoFundMe campaigns for individuals requesting donations for COVID-19 relief. Campaigns were identified by key word and manual review to categorize campaigns into “Traditional treatments,” “Alternative treatments,” “Business-related,” “Mandate,” “First Response,” and “General.” For each campaign, we extracted basic narrative, engagement, and financial variables. Among those that were manually reviewed, the additional variables of “mandate type,” “mandate stance,” and presence of COVID-19 misinformation within the campaign narrative were also included. COVID-19 misinformation was defined as “false or misleading statements,” where cited evidence could be provided to refute the claim. Descriptive statistics were used to characterize the study cohort.

**Results:**

A total of 30,368 campaigns met the criteria for final analysis. After manual review, we identified 53 campaigns (0.17%) seeking funding for alternative medical treatment for COVID-19, including popularized treatments such as ivermectin (n=14, 26%), hydroxychloroquine (n=6, 11%), and vitamin D (n=4, 7.5%). Moreover, 23 (43%) of the 53 campaigns seeking support for alternative treatments contained COVID-19 misinformation. There were 80 campaigns that opposed mandating masks or vaccination, 48 (60%) of which contained COVID-19 misinformation. Alternative treatment campaigns had a lower median amount raised (US $1135) compared to traditional (US $2828) treatments (*P*<.001) and a lower median percentile of target achieved (11.9% vs 31.1%; *P*=.003). Campaigns for alternative treatments raised substantially lower amounts (US $115,000 vs US $52,715,000, respectively) and lower proportions of fundraising goals (2.1% vs 12.5%) for alternative versus conventional campaigns. The median goal for campaigns was significantly higher (US $25,000 vs US $10,000) for campaigns opposing mask or vaccine mandates relative to those in support of upholding mandates (*P*=.04). Campaigns seeking funding to lift mandates on health care workers reached US $622 (0.15%) out of a US $410,000 goal.

**Conclusions:**

A small minority of web-based crowdfunding campaigns for COVID-19 were directed at unproven COVID-19 treatments and support for campaigns aimed against masking or vaccine mandates. Approximately half (71/133, 53%) of these campaigns contained verifiably false or misleading information and had limited fundraising success.

**International Registered Report Identifier (IRRID):**

RR2-10.1001/jamainternmed.2019.3330

## Introduction

The COVID-19 pandemic has resulted in tremendous health and financial consequences worldwide, with an estimated loss of 24 trillion dollars in the first year of the pandemic alone [1]. During the pandemic, many turned to crowdfunding to cover health expenditures, as they lost business revenue and were confronted with other new financial burdens; this increase has been similarly seen in the use of crowdfunding for other illnesses [2,3]. The initial increase in funding requests—150,000 campaigns (not all COVID-19 related) in March 2020 alone—were so dramatic that it prompted public comments to the US Congress from the GoFundMe website [4]. GoFundMe remains the largest crowdfunding service, and prior to the pandemic over one-third of the campaigns were used for medical purposes [5-8].

The pervasiveness of unproven or disproven theories about COVID-19—particularly the safety and efficacy of vaccination, nonpharmaceutical interventions such as masking, and potential treatments—poses a major and persistent public health challenge. The “infodemic”—defined as an “overwhelming, complex, and contradictory information... including fake news... about the origins of the virus... treatment options unsupported by data... and the life-saving vaccine”—has accompanied the COVID-19 pandemic [9]. The volume and pace of new information related to vaccination, masking, health care burnout, compassion fatigue, novel variants, and alternative treatments have changed the landscape and discussion around COVID-19 [10-12]. Furthermore, data continue to suggest that misleading COVID-19 information remains prevalent across search and social media platforms [13-18]. One study examined the use of GoFundMe to raise money for unproven COVID-19 prophylactic medications and suggested that GoFundMe be included as part of the conversation around COVID-19 misinformation [19]. As the world enters the third year of the pandemic, there have been media reports that GoFundMe has continued to be used to fundraise for COVID-19 misinformation campaigns, occasionally resulting in removal by GoFundMe [20,21]. While there is no stated policy on GoFundMe regarding COVID-19 misinformation, a press release noted that “Fundraisers raising money to promote misinformation about vaccines violate GoFundMe’s terms of service and will be removed from the platform” [22]. There are no recent data to examine the use of the platform as a means to disseminate unproven, disproven, or misleading COVID-19 information and to fundraise for these causes.

We conducted a comparative analysis of crowdfunding campaigns for COVID-19–related requests. We analyzed campaigns for financial outcomes to better understand the economic needs of this population and the ongoing social appetite for crowdfunding COVID-19–related issues. We specifically sought to evaluate fundraising seeking assistance for unproven, disproven, or misleading information, including alternative treatments not endorsed by public health agencies. In addition, we examined fundraising aimed at opposition to COVID-19 mitigation efforts, including masking and vaccination. Finally, we examined the prevalence of COVID-19 misinformation among these campaigns.

## Methods

### Data Source and Study Design

We performed a cross-sectional analysis of GoFundMe campaigns for individuals requesting donations for COVID-19 relief. All campaigns that were open or collected donations on GoFundMe from September 2021 to November 2021 were screened. Campaigns in which the story, title, or category included COVID-19 or the following key words were included: “COVID-19,” “Corona Virus,” “China Virus,” “SARS-CoV2,” “Pandemic,” “Right to Try*,” “Vit* D*,” “Hydroxychloroquine,” “Ivermectin,” “FrontLine COVID-19 Critical Care Alliance (FLCCC),” “Vaccine*,” “Mask*,” “Ventilator,” “Hospital*,” “Nitrous oxide (NOS),” “Melatonin,” “CoronaBox,” “Essential Oils,” “Herbal*,” “Homeopath*,” “Complimentary,” and “*Mandate*.” Key words were generated based on publicly available lists of frequently searched COVID-19–related “unproven” treatments and myths (Centers for Disease Control and Prevention, World Health Organization, Wikipedia, WebMD, Mayo Clinic, and Johns Hopkins) [23-27]. The list was reviewed and finalized in consultation with an expert author (ML). Asterix (*) indicates a variable to incorporate multiple suffixes or prefixes. For example, Homeopath* would collect searches in which Homeopathic or Homeopathy were included.

These key words were queried on the GoFundMe platform across 50 US states for 1200 searches (50 states x 24 key words = 1200 searches). Of note, the use of states improves search algorithm by limiting the individual search results in Python (Python Software Foundation) without changing the campaigns that were captured [8]. A custom Python programming language code was used to automatically retrieve information from publicly available campaign webpages. This search was conducted in November 2021. Manual review took place in February of 2022. This data collection methodology has been previously described [28,29].

### Categories and Manual Review

The initial study population was filtered by the same key words to ensure the inclusion of “COVID-19,” “Pandemic,” “Corona Virus,” “China Virus,” or “SARS-CoV2” in the title or story of the campaign in relation to the pandemic or its effect. Further key word categorization was performed to divide the selection into categories that were chosen a priori, such as “Alternative treatments,” “Business-related,” “First Response,” “Mandate,” “Traditional Treatments,” and “General” (Multimedia Appendix 1). “Alternative treatments” was defined by key words within the campaign (Multimedia Appendix 1), which focused on the use of unproven or complementary therapies for COVID-19 (eg, “The specific goal of this fundraiser is to support [treatment] with Ivermectin”). Moreover, “Business-related” was defined by key words (Multimedia Appendix 1) that focused on funding for lost income or commercial revenue (eg, “Covid was devastating for my businesses and I wasn't eligible for jobkeeper so I've been on a bit of a knife edge for the last year”). “First Response” was defined by key words (Multimedia Appendix 1) that focused on funding for COVID-19 first responders, mission trips, or community organizations (eg, “Unfortunately, these hardworking men and women [nurses] don't have the necessary protection they need to stay safe as they save lives so that they can continue to save lives”). “Mandate” was defined by key words (Multimedia Appendix 1) that focused on funding for legal relief from COVID-19–related restrictions (eg, “Breathe Free Colorado was founded to stop the unconstitutional mask mandate created by Governor through executive order”). “Traditional Treatments” was defined by key words (Multimedia Appendix 1) that focused on funding for COVID-19 hospital or other health expenditures (eg, “Mickey has contracted covid19 and is in the UIHC in Iowa City on a ventilator and very sick. Mickey was the household income so they're needing help plus hospital Bill's are piling up. Please help if you can”). All other campaigns that did not fall into the established categories based on key word were defined as “General.” A hierarchy was established such that campaigns in which multiple key words were present were preferentially assigned to (1) “Alternative Treatment,” (2) “Mandate,” (3) “Traditional Treatments,” (4) First Response,” and (5) “General.” This hierarchy was chosen a priori, knowing categories 1 and 2 would be manually reviewed.

All campaigns identified by key word in the categories “Mandate” and “Alternative Treatment” were manually reviewed. Manual review confirmed that the campaign was seeking funding relief for the alternative treatment or mandate-related expenses and confirmed the web-scraped variables (eg, funding goal and total funds raised) were accurate and up-to-date. Furthermore, manual review of the “Mandate” category facilitated further analysis of whether the mandate-related campaigns were pro- or antimask and pro or antivaccine. Manual review was also performed for a random sample of 100 campaigns from each other category (“Business-related,” “First Response,” “Mandate,” “Traditional Treatments,” and “General”) as a similar sensitivity analysis. Campaigns were permitted to be moved from one category to another or excluded on manual review.

**Figure 1:**
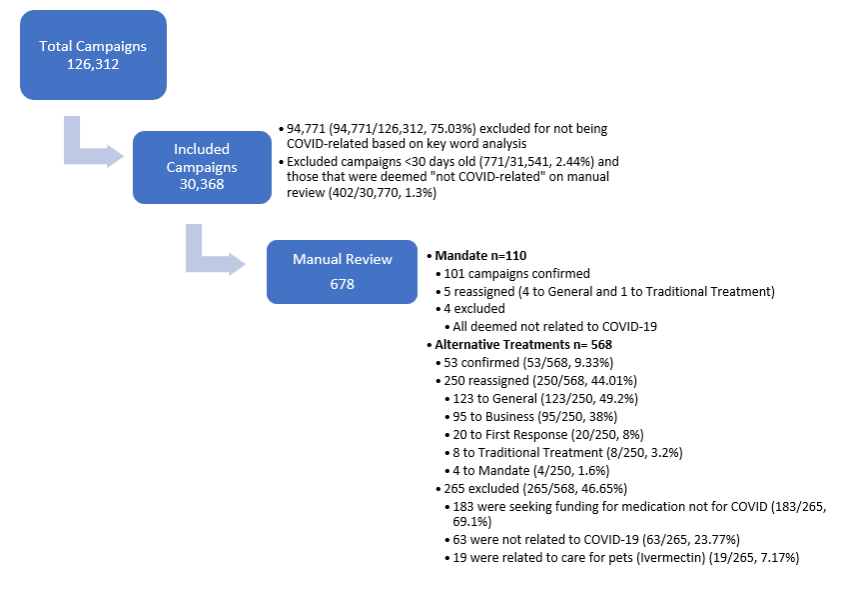
Inclusion of COVID-19–related campaigns.

Manual review was also undertaken to capture campaigns containing explicit COVID-19 misinformation. COVID-19 misinformation for the purposes of this study adapted the standard definition of “false or misleading information meant to deceive” [30] to include only those claims where direct cited evidence could be provided to refute a claim. For each misleading statement, a reference is provided to directly refute this claim. Vague claims or those that could not be possible to prove false were not considered misinformation. For example, a campaign claiming individual side effects from the vaccine, which could not possibly be verified, would not be coded as containing misinformation. Similarly, charged language around vaccination (eg, “jab of Satan”) was not considered misinformation. Statements such as “masks cause harm” or “children cannot get COVID-19” were considered misinformation, and references were provided demonstrating the claims as false. Finally, the manual review stated if the campaign was withdrawn. The full review process is summarized in Figure 1. The design and reporting of this study adhered to the guidelines of the Strengthening the Reporting of Observational Studies in Epidemiology (STROBE) statement (Multimedia Appendix 2) [31].

### Variables

For each campaign, basic narrative features, engagement metrics, and financial variables were extracted. We examined campaign titles, narratives, creation date, number of social media shares, campaign goal amount, number of donations, and amount of funding raised. Among those that were manually reviewed, the variables of “Mandate Type,” “Prorestriction,” and “Antirestriction” were also included. Campaigns were also reviewed for COVID-19 misinformation. As described above, this adhered to the strict definition of verifiably false information included in the title, description, or story of the included campaign. Within the “Alternative Treatment” category, the individual drug or treatments were reviewed and included in the analysis.

### Statistical Analysis

We used descriptive statistics to characterize the study cohort. Continuous variables skewed distribution and were reported as median (interquartile range). Campaign success was defined as those that received funding amounts equal to the declared goal or higher. Campaigns with no funding goal were considered neither successful nor unsuccessful. Campaigns that were not open for at least 30 days at the time of data collection were excluded. We reported the proportion of campaigns deemed successful as well as the median percentile of funding goal received. Characteristics of successful and unsuccessful campaigns were compared using the chi-square or Fisher exact tests for categorical variables (campaign success, median goal, median raised, median donors, median donations, and campaign type) or Mann Whitney *U* test for continuous variables (donors, donations, total amount raised, total campaign goal, photos, updates, comments, shares, and followers). In a similar fashion, we compared campaigns in the “Traditional Treatments” category to those in the “Alternative Treatments” category, and we compared those supporting vaccine or mask mandates to those opposing vaccine or mask mandates within the “Mandates” category. We used Stata 17 (StataCorp), and we considered a 2-sided *P* value of <.05 as statistically significant.

### Ethics Approval

The data collected from GoFundMe were deidentified and did not qualify under the University of California Institutional Review Board (IRB) as human subjects research. Therefore, IRB approval was not sought, and appropriate IRB self-certification was completed and maintained per University of California San Francisco policy.

## Results

### Campaigns

A total of 126,695 campaigns were identified for analysis, 126,312 (99.7%) of which were active from April 2011 to November 2021. Of those, 30,368 (24.0%) campaigns met the criteria for final analysis (Figure 1). Moreover, 6444 campaigns were seeking aid for medical costs associated with COVID-19. The majority of these (6391/6444, 91.2%) were seeking aid for traditional treatments including hospital or intensive care unit care (4285/6444, 66.5%), medical bills or hospital bills (1569/6444, 24.3%), remdesivir (63/6444, 0.98%), monoclonal antibody treatment (16/6444, 0.25%), and home oxygen (13/6444, 0.20%; Multimedia Appendix 1). Based on key word, there were 568 campaigns assigned to alternative treatments. After excluding those classified as “General,” “Business,” “First Response,” Traditional Treatment,” and “Mandate,” there were 53 campaigns (53/30,368, 0.17%) that were expressly seeking funding for unproven or alternative treatments for COVID-19 (Table 1). These campaigns requested donations including stories asking for herbal remedies (17/53, 32%), ivermectin (14/53, 26%), hydroxychloroquine (6/53, 11%), and vitamin D (4/53, 7.5%).

**Table 1:** Campaign characteristics and predictors of campaign success.

Category	Values			
	All	Unsuccessful^a^	Successful	*P* value^b^
Total, n (%)	30,368 (100)	25,502 (86.9)	3847 (13.1)	.003
Alternative treatments, n (%)	53 (0.17)	44 (88)	6 (12)	—^c^
Mandate, n (%)	96 (0.32)	90 (95.7)	4 (4.3)	—
First response, n (%)	3260 (10.7)	2691 (86.5)	421 (13.5)	—
Traditional treatments, n (%)	6391 (21)	5270 (85.7)	883 (14.4)	—
Businesses, n (%)	415 (1.4)	353 (87.4)	51 (12.6)	—
General, n (%)	20,153 (66.4)	17,054 (87.3)	2482 (12.7)	—
Number of followers, median (IQR)	27 (4-75)	21 (2-62)	81 (39-172)	<.001
Number of donations, median (IQR)	21 (2-58)	16 (1-48)	63 (30-134)	<.001
Number of donors, median (IQR)	20 (2-55)	15 (1-46)	60 (29-127)	<.001
Ratio donations:donors, median (IQR)	1.01 (1-1.05)	1.006 (1-1.05)	1.019 (1-1.05)	<.001

^a^Some campaigns did not have funding goal despite remaining open. This accounts for the small number missing between the total n and those included in the campaign success columns.

^b^Statistical tests comparing successful vs unsuccessful campaigns.

^c^Not available or appropriate.

### Alternative Versus Traditional Treatment

**Figure 2:**
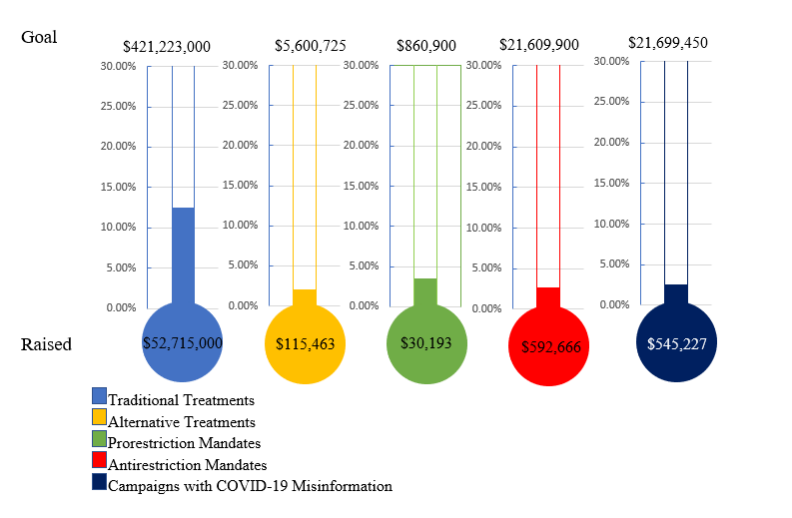
Percent of total funding goal reached in US dollars.

Among the 53 campaigns identified as seeking funds for unproven COVID-19 treatment, the median fundraising goal (US $10,750) was higher than campaigns seeking money for traditional treatment (US $10,000), and fewer alternative campaigns met their stated goal (9/53, 16.9%) compared to traditional (1121/6391, 17.5%; *P*=.92). Alternative treatment campaigns had a significantly lower median amount raised compared with traditional treatments (US $1135 vs US $2828, respectively; *P*<.001) and a lower median percentile of target achieved at 11.9% for alternative compared to 31.1% for traditional treatments (*P*=.003). Taken as an aggregate, traditional treatments raised 12.5% of the total requested funding goal while alternative treatments reached only 2.1% (Table 2; Figure 2). On manual review, the 6 (6/53, 11%) campaigns in the alternative treatment that met their goal were substantively different from the unsuccessful campaigns. Moreover, 2 (2/53, 4%) campaigns were written for family members who were severely ill in the intensive care unit requesting funds to add alternative treatments to traditional as “last resorts.” An additional 2 (2/53, 4%) campaigns were seeking funding for herbal remedies to help whole communities—Navajo nation and an unspecified “ancient folk” community. Only 2 (2/53, 4%) successful campaigns were written by the individual seeking funds, and both cited multiple other hardships and medical comorbidities in addition to asking for funding for alternative treatment (ivermectin in both cases). Another successful campaign was seeking funding for a study to evaluate the efficacy of vitamin D for treatment and prevention of COVID-19. Interestingly, despite its success, this campaign was withdrawn during the period of manual review. Finally, the highest grossing successful and unsuccessful alternative treatment campaigns (US $12,555 donated from US $100 asked and US $11,684 donated from US $30,000 asked) were seeking funding for frontline medical personnel, including the use of vitamin D and ivermectin for the treatment and prevention of COVID-19.

Alternative treatments contained COVID-19 misinformation in 23 (43%) campaigns including advocacy on alternative treatments over vaccination or traditional treatments (n=18, 34%), claims that the pandemic is not real or is a conspiracy (n=2, 4%), and false claims about the safety or efficacy of the vaccine (n=3, 7%). From the manual review of the subset (n=100) of traditional treatments, none of them contained COVID-19 misinformation. A total of 5 (5/53, 9.4%) alternative treatment campaigns were withdrawn by the manual review period compared to 1 (1/100, 1%) in the traditional group (Table 3).

**Table 2:** Comparison of traditional and alternative treatment campaigns.

Characteristics		Alternative treatments	Traditional treatments	*P* value	
Total campaigns		53	6391	—^a^	
Success, n (%)		9 (16.9)	1121 (17.5)	.92	
Total amount raised (US $)		115,463	52,715,762	—	
Total amount goal (US $)		5,600,725	421,223,293	—	
Total achieved (%)		2.06	12.5	—	
**Campaign features**					
	Goal (US $), median (IQR)	10,750 (2700-30,000)	10,000 (5000-23,000)	.81	
	Funds raised (US $), median (IQR)	1135 (50-2975)	2828 (665-7788)	<.001	
	Funds raised^b^ (%), median (IQR)	11.9 (0.01-38.9)	31.1 (6.8-74.9)	.03	
	Photos, median (IQR)	1 (1-3)	1 (1-2)	.76	
	Number of updates, median (IQR)	0 (0-3)	1 (0-2)	.78	
	Number of comments, median (IQR)	0 (0-2)	1 (0-3)	.009	
	Number of donations, median (IQR)	17 (1-36.5)	35 (10-85)	.001	
	Number of donors, median (IQR)	16.5 (1-32)	33 (9-81)	.001	
	Ratio donations:donors, median (IQR)	1.017 (1-1.09)	1.014 (1-1.047)	.5	
	Number of shares, median (IQR)	26.5 (0-219)	113 (5-398)	.007	
	Number of followers, median (IQR)	22 (1-47)	45 (13-109)	.001	

^a^Analyses not performed or not appropriate.

^b^Total funds raised divided by requested. This only applied to campaigns with a funding goal.

**Table 3:** Summary of unproven “alternative treatment” campaigns.

Alternative treatments	All, n (%)	Successful n (%)	Funds raised (US $), n (% of requested)	Funds requested (US $)	COVID-19 misinformation, n (%)	Withdrawn, n (%)
Total	53 (100)	7 (13.2)	115,463 (2.06)	5,600,725	23 (43)	5 (9.4)
Herbal or homeopathic remedies	17 (32)	3 (17.6)	20,410 (0.44)	4,606,475	7 (41)	2 (3.7)
Ivermectin	14 (26)	1 (7.1)	25,615 (10.7)	238,800	11 (79)	1 (1.8)
Hydroxychloroquine	6 (11)	2 (33.3)	42,728 (17.5)	242,100	0 (0)	1 (1.8)
Vitamin D	4 (7.5)	0 (0)	4478 (12.4)	36,000	3 (75)	1 (1.8)
Meditation or functional	2 (4)	0 (0)	2063 (0.81)	252,700	0 (0)	0 (0)
Naturopathic	2 (4)	0 (0)	11784 (23.4)	50,250	1 (50)	0 (0)
Air purification	1 (2)	0 (0)	130 (0.13)	100,000	0 (0)	0 (0)
Essential oils	1 (2)	0 (0)	1135 (9.9)	11,500	0 (0)	0 (0)
Lifestyle	1 (2)	0 (0)	0 (0)	15,000	0 (0)	0 (0)
Music	1 (2)	0 (0)	0 (0)	30,000	0 (0)	0 (0)
Native American remedies	1 (2)	1 (100)	918 (153)	600	0 (0)	0 (0)
Melatonin	1 (2)	0 (0)	1880 (15.6)	12,000	0 (0)	0 (0)
Soaps	1 (2)	0 (0)	1197 (66.5)	1800	0 (0)	0 (0)
Vitamin C	1 (2)	0 (0)	3125 (90)	3500	1 (100)	0 (0)

### Mandates

A total of 96/30,368 (0.32%) campaigns were created to fund political positions in response to mask or vaccine mandates. Of these 93 campaigns, 80 (83%) were created to oppose restrictions (against requiring masks or vaccines; the most frequent mask mandates were in schools). The vast majority of campaigns did not meet funding goal (90/96, 86.9%), and the median funding percentage (proportion of goal reached) was 14.2%.

The median goal for campaigns was significantly higher (US $25,000 vs US $10,000) for campaigns opposing mask or vaccine mandates (*P*=.04). Almost all (14/16, 87.5%) campaigns supporting restrictions (prorestriction) were in support of reinstating mask mandates, particularly in schools in areas where the mandate had been or was going to be lifted (Table 4). Category, followers, donations, and ratio of donations:donors were significantly associated with campaign success (Table 1). Among the campaigns seeking changes to health care mandates, one campaign seeking broad legal challenges against a state governor obtained US $31,730 (42.3%) of the US $75,000 requested. The remaining 6 campaigns obtained US $622 (0.2%) out of US $410,000. This is in contrast with campaigns seeking to re-enforce masking in schools which raised $37,247 (4.9%) of $761,400.

Among the 80 campaigns requesting support for antirestriction positions, 48 (60%) contained verifiably false claims regarding COVID-19 and COVID-19 vaccination. This included claims that masks were ineffective or dangerous (n=18, 22.5%), children are unaffected by COVID-19 (n=10, 12.5%), COVID-19 is mild or similar to the common cold (n=10, 12.5%), individuals would be better to seek nonvaccine prevention (n=6, 7.5%), equivocal efficacy between various unproven COVID-19 treatments (n=4, 5%), the pandemic is not real or is a conspiracy (n=2, 2.5%), and the vaccine is not safe or effective (n=1, 1%; Table 5). Comparatively, the small minority of campaigns seeking to reimpose restrictions (16) contained no COVID-19 misinformation.

**Table 4:** Mandate-related campaigns.

Characteristics of the campaigns			Prorestriction^a^ (n=16)		Antirestriction^b^ (n=80)		*P* value
Median goal in US $, n (IQR)			10,000 (2000-25,000)		25,000 (10,000-75,000)		.04
Median raised in US $, n (IQR)			283 (0-3120)		210 (0-5131)		.82
Successful campaigns, n/N (%)^c^			2/15 (13.3)		2/79 (2.5)		.12
Number of donations, median (IQR)			4 (0-34)		5 (0-83.5)		.65
Number of donors, median (IQR)			4 (0-33)		5 (0-79.5)		.66
**Mandate type, n (%)**							.007
	Mask	14 (87.5)		35 (43.8)			
	Vaccine	2 (12.5)		37 (46.3)			
	All restrictions	0 (0)		8 (10)			
**Mandate setting, n (%)**							.01
	School	11 (68.8)		32 (40)			
	Job	0 (0)		22 (27.5)			
	Health care	0 (0)		7 (8.8)			
	None or general	5 (31.3)		19 (23.8)			
Contains COVID-19 misinformation, n (%)			0 (0)		48 (60)		<.001
**Funds (US $) received of total requested, n/N (%)**							—^d^
	Total	30,193/860,900 (3.51)		514,584/21,419,900 (2.4)			
	Mask	29,626/833,400 (3.55)		403,964/11,583,000 (3.49)			
	Vaccine	567/27,500 (2.06)		110,620/9,836,900 (1.12)			

^a^Prorestriction indicates campaigns that were seeking to reinstate, strengthen, or support existing mandates on masking or vaccination. A recurring example was parents seeking support to reinstate mask mandates in schools that had lifted mask mandates.

^b^Antirestriction indicates campaigns that were seeking to remove mandates that required masking or vaccination.

^c^Some campaigns did not have funding goal despite remaining open. This accounts for the small number missing between the total n and those included in the campaign success columns.

^d^Not available or appropriate.

**Table 5:** Description of COVID-19 misinformation in GoFundMe campaigns.

Misinformation	Value (N=71^a^), n (%)	True statement with reference
[Alternative Treatment] is equivalent to traditional treatments or can replace vaccination.	22 (31)	“There is no evidence to support the efficacy of alternative treatments. There is decreased COVID-19 transmission and hospitalization rate among vaccinated individuals” [32].
Masks are ineffective or dangerous.	18 (25)	Masks are effective in decreasing the spread of COVID-19 [33].
Children cannot get COVID-19 or are not seriously affected.	10 (14)	“Children are less likely to experience severe COVID-19 compared to adults, but still have concerning rates of hospitalization and serious illness” [34].
COVID-19 is not severe or is equivalent to the common cold.	10 (14)	“There is a clinical spectrum including severe illness and death resulting from COVID-19 infection” [35].
Government is forcing an unsafe or experimental vaccine	6 (8)	“Mandates are in place for certain professions” [36]. “Data on vaccine safety is robust” [37].
Vaccination results in severe side effects.	4 (6)	“Vaccine side effects are rare and overwhelmingly mild” [37,38].
The pandemic is fake or a conspiracy.	4 (6)	“There are numerous and changing conspiracies about the origins and reality of the COVID-19 pandemic” [39].

^a^Some campaigns contained more than one verifiably false statement, so the sum of categories is greater than total campaigns containing false statements.

## Discussion

### Principal Results

There is a minority on the social media site GoFundMe seeking financial support for fringe beliefs regarding COVID-19. Among the small number of campaigns seeking assistance for alternative treatments and to oppose mandates, the majority (71/133, 53%) contained verified false information about COVID-19. These findings are novel in the literature for crowdfunding and verify the trends occurring amid the infodemic across other social media sites and the popular reporting on GoFundMe [20,21,40-42].

A small minority of web-based crowdfunding campaigns for COVID-19 on the GoFundMe platform explicitly sought funding for unproven or disproven medical treatments, as well as opposition to public health interventions such as masking (Table 5). Crowdfunding campaigns for alternative COVID-19 treatments and antimasking generated fewer views and less funding compared to campaigns seeking funding for traditional medical treatment. Interestingly, the number of campaigns that reached their stated goal was similar between alternative treatments and traditional treatments. Despite the similarity in “successful campaigns,” the funds generated as a percentage of the total requested between alternative (US $115,000/$5,600,000, 2.06%) and traditional treatments (US $52,715,000/$421,223,000, 12.5%) were substantially different. The discrepancy indicates that a small number of campaigns are able to generate significant funding while the majority fail to generate even a small percentage of the requested funds. These findings highlight the pervasiveness of unproven or disproven information about COVID-19, and the complex media through which this information continues to propagate.

This study is the first to examine the use of crowdfunding for positions on COVID-19–related mandates. We found that campaigns seeking funding for positions on mask and vaccine mandates rarely met funding goals. The majority of campaigns were in opposition to COVID-19 restrictions. In total, 48/80 (60%) of the antirestriction campaigns contained COVID-19 misinformation compared to none in the prorestriction category. Overall, there seemed to be very little interest in funding campaigns seeking to take political or legal action against mandates, particularly compared to the relative success seen in campaigns seeking medical care. An interesting subcategory consisted of the 7 campaigns in opposition to COVID-19 restrictions for health care workers, none of which achieved success, having raised very minimal funding despite asking for sizable donations. Among the campaigns written by or for those in the health care industry, all but one contained false or misleading claims about COVID-19. Health care workers continue to represent a challenging demographic, with significant regional variation in vaccination rates and opinions on COVID-19 treatments [43,44]. The lack of support and the small minority of health care workers seeking assistance may suggest increased COVID-19 awareness, limited appetite to fund such endeavors, or both.

The overall success rate of COVID-19–related campaigns approached 14%, indicating a sizeable and persistent gap between the funds raised and the funding needs of individuals still affected by COVID-19 [29]. Compared to a similar analysis by Saleh et al [2], in the first months of the pandemic (March 2020), this study shows a similar rate of success as measured by campaigns reaching their goal. In contrast to that study, we found a much higher median goal (US $10,000 vs US $5000) with a higher median amount raised (US $2,808 vs US $930). Two years into the pandemic, there is still support for campaigns at approximately the same level previously noted in the literature. Despite this continued support, there were substantial differences in the types of campaigns that received funding.

### Limitations

Our study has several limitations. A major limitation is the singular time frame when the campaigns were collected. The goal was to obtain a snapshot of the landscape of COVID-19 crowdfunding, but only active or recently closed campaigns were captured. As the date range (2011-2021) suggests, some of these campaigns had been active for a long time while others were recently open. The older campaigns (including those that preexisted COVID-19) were subject to the same screening for relevance. We intentionally excluded campaigns open for <30 days to minimize this limitation. Conversely, it is possible that some of the most successful campaigns open and close quickly after meeting funding goals. This was not demonstrated in the sensitivity analysis when we reviewed campaigns open less than 30 days. Moreover, the description of “alternative treatments” has been presented in a singular study, and we take our definition from that study [45]. We sought to capture the broad interpretation of medical treatments that have not been adopted as standard of care in the treatment of COVID-19. Data are always evolving, and some or all of these treatments may become part of that standard in the future. Despite these limitations, this is the first cross-sectional analysis of COVID-19 misinformation among GoFundMe campaigns and the first study to examine the crowdfunding for political and legal challenges to COVID-19 mandates. Furthermore, it adds to the growing body of literature on social networking and crowdfunding for alternative treatments to COVID-19.

### Conclusions

There was a small minority of crowdfunding campaigns seeking assistance for unproven alternative COVID-19 treatments, about half of which contained COVID-19 misinformation and very few of which were successful. Additionally, the majority of campaigns seeking funding for legal or political action against COVID-19 mandates contained COVID-19 misinformation and were unpopular and underfunded. Crucially, these data also raise concerns for the moral and ethical implications of the funds raised; where these monies used to fund the purposes stated or where they meant to capitalize on often politically charged beliefs? Our findings have implications for how individuals and organizations seek and obtain funding and add to the growing commentary on the ethical challenges associated with social media, COVID-19, and misinformation.

## Appendix

Multimedia Appendix 1 Key word categorization and search terms for each category.

Multimedia Appendix 2 Strengthening the Reporting of Observational Studies in Epidemiology (STROBE) checklist.

## Abbreviations

IRB: Institutional Review Board
STROBE: Strengthening the Reporting of Observational Studies in Epidemiology
