# Supplementary material for: COVID-19 Misinformation and Social Network Crowdfunding: Cross-sectional Study of Alternative Treatments and Antivaccine Mandates
Source: J Med Internet Res. 2022 Jul 27;24(7):e38395. doi: 10.2196/38395 (PMC9337619; doi:10.2196/38395)
Supplement: Multimedia Appendix 1 [file jmir_v24i7e38395_app1.docx]

Appendix 1: Search terms for each category

**Alternative treatments**

Andrographis paniculata

Arsenicum*

artemisia plant

Bananas

betel

bittergourd

cannabis

carbolic soap

Chlorine dioxide

Chloroform

*chloroquine

chlorous acid,

Cocaine

Colchicine

Colloidal Silver

creams

datura

dietary supplements

durian

Essential Oils

Exercise

Exorcism

FrontLine COVID-19 Critical Care Alliance (FLCCC)

Garlic

Globuli

hand or hair dryers

Hand sanitizer

Happy Science,

Homeopath*

honey

hydrogen peroxide  nebulizer

Interferon*

Interluekin*

Ivermectin

Lemon*

loló

Mango

Meditation

Methanol

Methylene chloride

Monoclonal*

oleandrin

Onions

Pendants

perfume

Plants

Protease inhibitor

Right to Try

Red soap

Remdesivir

saltwater

Saunas

Shooting stars

Shrines

Shuanghuanglian

Shut Out

sodium chlorite

sunlight

Tamiflu

Tinospora crispa

Toothpastes

Traditional* Medicine

treatment packs

turmeric

UV-C

violet oil

vitamin C

vitamin D

Wudu

Zinc

*Antibody

*herb*

*peppers

*steroid

*Urine

**Business-related**

*Business

Family-owned*

Lost*

Restaurant

Self-employed
Store*

*Wages

**First Response**

*Donation

Fire*

Police*

Emergency Medical Technician (EMT)

Nurse*

**Mandate**

Mask Mandate

Healthcare Mandate

School*

Vaccine Mandate

Vaccine Requirement

**Traditional treatments**

*Bill

Hospital*

Intensive Care/ICU

Ventilator

Note: Many approved treatments are listed under alternative. This was to facilitate manual review.

**Other**

Note: No keywords beyond those mentioned to include campaign in overall COVID-related.
